# Curcumin alleviated oxidation stress injury by mediating osteopontin in nephrolithiasis rats

**DOI:** 10.1590/acb380223

**Published:** 2023-05-01

**Authors:** Jian-jun Huang, Xu-ping Yao, Ping Zhang, Zhi-ling Lou, Hong-gang Qi, Hou-meng Yang, Guo-bin Weng

**Affiliations:** 1 Ningbo University – Ningbo Urology and Nephrology Hospital – Department of Urology – Ningbo, China.

**Keywords:** Curcumin, Glycol, Nephrolithiasis, Osteopontin, Stress

## Abstract

**Purpose:**

To explore the role and mechanism of curcumin (Cur) in reducing oxidative stress damage in rats with nephrolithiasis induced by ethylene glycol (EG).

**Methods:**

Thirty male rats were divided into normal control, model, positive (10% potassium citrate), Cur-10 (10 mg/kg curcumin) and Cur-20 (20 mg/kg curcumin) groups.

**Results:**

The results of kidney tissue section stained by hematoxylin-eosin and von Kossa showed that curcumin treatment can inhibit the formation of kidney stones. The biochemical test results showed that the urea (Ur), creatinine (Cr), uric acid (UA), inorganic phosphorus and Ca^2+^ concentrations in urine decreased after being treated with curcumin. There were significant differences between different doses of curcumin (P < 0.05). Compared with the Cur-10 group, Cur-20 had a more significant inhibitory effect on malondialdehyde (MDA) (P < 0.05). In addition, reverse transcription polymerase chain reaction (PCR) detection and immunohistochemical results indicated that the osteopontin (OPN) in the kidney was significantly reduced after curcumin treatment.

**Conclusions:**

Curcumin could reduce the oxidative stress damage caused by EG-induced kidney stones.

## Introduction

Nephrolithiasis is a common urinary system disease. The annual prevalence of nephrolithiasis worldwide is 3–5%, which severely impacts human health^1^. More than 80% of kidney stones are composed of calcium oxalate, with calcium oxalate hydrate as the primary form^2^^,^ 3. The formation of kidney stones involves pathophysiological processes such as oxidative stress, inflammation, apoptosis, fibrosis, and autophagy^4^^,^ 5. At present, the treatment of nephrolithiasis is mainly through minimally invasive surgery to remove stones^5^^,^ 6, while its high recurrence rate is still very high^5^^,^ 7 ^-^ 9. Compared with surgical treatment, drug treatment of kidney stones is safer and more convenient, which may also prevent the recurrence of stones^10^. Therefore, it is important to develop drugs that can safely and efficiently treat kidney stones.

Curcumin (Cur) is a polyphenolic substance isolated from the turmeric rhizome. Curcumin is the main active component of turmeric, and it has various functions, including antioxidant^11^, anti-inflammatory^12^, antifibrosis^8^, antibacterial and antitumor activities^13^, and protection from oxidative damage in the kidney^14^. Animal models of oxidative damage to kidney tissue and kidney stones have proven that curcumin can reduce the expression of inflammation-related factors, and induce anti-inflammatory factors to inhibit the activation of the MAPK/ERK, TGF-β/Smad, and PPAR-γ pathways^11^^,^ 14 ^,^ 15. In addition, curcumin could induce the antioxidant response in the kidney, and nuclear factor Nrf2 to play a regulatory role, inhibit mitochondrial dysfunction, reduce inflammation and prevent oxidative stress damage^15^^,^ 16. Studies have shown that curcumin exhibits important renal protection and stone suppression effects in kidney diseases^11^^,^ 17. However, studies on the effects of curcumin about oxidative stress state during the process of kidney stone formation and its underlying mechanism are relatively rare.

Ethylene glycol (EG) is the precursor of oxalic acid. After being absorbed in the body, it is quickly distributed in blood and tissue fluid, and, finally, oxalic acid is formed in the liver. It is often used as a stone attractant for kidney calcium oxalate stone models. NH_4_Cl can acidify the urine and reduce calcium oxalate stones in the urine. The combination of EG and NH_4_Cl can induce calcium oxalate stones in the kidney^18^^,^ 19. Citric acid is a potent calcium ion chelator that can reduce the activity, concentration, and saturation of calcium ions, reducing the formation of calcium salt crystals. It can also inhibit the precipitation of calcium oxalate and calcium phosphate crystals, prevent the formation of stones, the growth and aggregation of nuclei, and reduce the deposition of stones. It is usually used as a clinical drug for the treatment of kidney stones^5^^,^ 15 ^,^ 20. This study further explored of the effect of curcumin in the EG-induced rat kidney stone model to elucidate the mechanism by which curcumin alleviate oxidative stress damage in nephrolithiasis rats.

## Methods

### Reagents and instruments

Curcumin (C805204), potassium citrate (P816198), EG (E808735), and NH_4_Cl (A801304) were all purchased from the Macklin Biochemical Technology Co., Ltd. (China, Shanghai). Malondialdehyde (MDA) assay kit (A003-1-2) and superoxide dismutase (SOD) assay kit (A001-3-2) were all from Nanjing Jiancheng Bioengineering Institute (Nanjing, China), osteopontin (OPN) antibody (SAB4501013, Sigma-Aldrich, USA). The fluorescence quantitative polymerase chain reaction (PCR) detection kit (0513471) and reverse transcription kit (0512931) were all from Novoprotein Co., Ltd. (Shanghai, China). Automatic biochemical analyzer (CM-800 Hitachi, Japan), real-time fluorescent quantitative PCR instrument (7500 Fast, Thermo Scientific, USA), optical microscope (DM500, Leica, Germany), microplate reader (Multiskan MK3, Thermo Scientific, USA).

### Animals

The study was approved by the Experimental Animal Ethics Committee of Ningbo University and supervised throughout the process.

A total of 50 male specific-pathogen free Sprague Dawley rats (6–8 weeks, body weight, 200~250 g) were obtained from Shanghai Jiesijie Laboratory Animal Co., Ltd. (Shanghai, China). Rats were maintained in the following experimental conditions: a 12-h light-dark cycle; a maintained temperature of 20–25 °C with a relative humidity of 40–70%. The animal ethics clearance was approved by the Animal Ethics Committee of the School of Medicine of Ningbo University.

### Nephrolithiasis models and treatment

Animal models were established according to Wesson’s method with modification^21^. They were randomly divided rats into five groups: control, model, positive group, Cur-10 (10 mg/kg curcumin), Cur-20 (20 mg/kg curcumin). After all, the rats were randomly divided into cages and adapted for 1 week. All groups of rats except the control group were given intragastric stone attractant every week: 2% NH_4_Cl, according to the dose of 10 mL/kg, 6 times a week for 4 weeks, and 1% EG was given in the drinking water every day. The control group was fed with distilled water every day. Drug intervention was started at the beginning of the third week. For the Cur-10 and Cur-20 groups, a suspension of curcumin was given subcutaneously injected at the back of the neck, while the positive control group was given 2% NH_4_Cl (solution in 20% potassium citrate).

### Specimen collection

On the day before the end of the experiment, 3 rats were randomly selected from each group and placed them in a metabolic cage, the urine samples were collected and stored at –80 °C from the rats within 12 h. When the experiment was over, the rats were anesthetized with 2.5% phenobarbital sodium (45 mg/kg, i.p.). The blood was collected from the abdominal aorta, and serum was separated by centrifugation at 3000× g for 5 min, aliquoted and stored at –80 °C. The rat kidneys were stripped and weighed. The left kidneys were fixed in paraformaldehyde and embedded in paraffin for staining and immunohistochemistry. Kidney index calculation formula: Kidney index = (2 × kidney weight/body weight) × 100, unit: g. The test was repeated three times for each sample.

### HE staining

Rat kidney tissue (1 × 1 × 0.5 cm^3^, n = 6) was treated with 4% paraformaldehyde for 24 h and embedded in paraffin after dehydration. The samples were cut into 5 μm pieces. Tissue was dewaxed in water, stained with hematoxylin and eosin (HE), and dehydrated sealed with neutral gum. Finally, the sample was photographed with an optical microscope (Leica). The test was repeated three times for each sample.

### Von Kossa dyeing

The paraffin sections of kidney tissue paraffin stions from the five groups (n = 6) were immersed in 5% silver nitrate, irradiated with ultraviolet light for 45 min, washed with water for approximately 6–7 min, and treated with 5% sodium thiosulfate aqueous solution for 2 min. Then, the tissue sections were soaked in water for 5 min, dyed with 0.1% nuclear fast red for about 5 min, washed with water for 5–10 s, dehydrated and mounted, then observed with an optical microscope.

### Oxidative stress index analysis

The right kidney tissue of the five groups (n = 6) was washed twice with precooled phosphate buffered saline (PBS) solution. Then 0.5 g tissue was placed in a tissue homogenizer, 5 mL precooled PBS was added, and tissue homogenate was prepared. After centrifugation, the culture supernatant was collected for MDA and SOD detection. The detailed procedure was performed according to the kit instructions. Finally, a microplate reader was used to read the OD value. The test was repeated three times for each sample.

### Biochemical indicator detection

An automatic biochemical analyzer detected the contents of urea (Ur), creatinine (Cr), uric acid (UA), and calcium (Ca^2+^) in urine, the contents of UA, inorganic phosphorus (IP), Ca^2+^, SOD, MDA in the right kidney and Ur, Cr, UA, IP, Ca^2+^ content in serum of the five groups (n = 6) . The test was repeated three times for each sample.

### Immunohistochemistry

The slices were placed in citrate buffer (pH = 6.0, 121 °C), added 3% H_2_O_2_ solution and incubated in the dark, and blocked with 1% BSA solution. After antigen retrieval, the primary antibody (1:100) was added and incubated overnight at 4 °C. Then, the secondary antibody (1:100) was added and incubated at room temperature for 1 h, added the diaminobenzidine color developing solution, and the sections were counterstained with hematoxylin for 30 s. Finally, the sections were dehydrated, and the film was mounted with neutral gum to detect the expression and distribution of OPN. ImageJ was used to grayscale the photographies, three parts of each group of field-of-view photos were randomly selected, and the gray value was calculated for quantitative statistics. The test was repeated three times for each sample.

### Quantitative reverse transcription-PCR for detecting mRNA expression levels

The primer for the OPN gene was designed through with National Center for Biotechnology Information (NCBI) (https://www.ncbi.nlm.nih.gov/), and β-actin was used as the internal reference. Total RNA was extracted by the TRIzol method, and the RNA concentration was detected by Nanodrop (Thermo Fisher Scientific, USA). Then, the target complementary DNA was obtained by reverse transcription, and fluorescence quantitative PCR reaction was performed according to NovoStart SYBR qPCR SuperMix Plus. The conditions were as follows: predenaturation, 95 °C, 2 min. PCR reaction, 95 °C 20 s, 55 °C 30 s, 72 °C 40 s, 40 cycles. The fluorescence signal was detected at 72 °C and the relative gene expression was calculated based on the cycle threshold (CT) value. The primer sequences were: Rat-OPN-F: CCA GCC AAG GAC CAA CTA CA, Rat-OPN-R: CTG CCA AAC TCA GCC ACT TTC; Rat β-actin-F: CCG TGA AAA GAT GGA CCC AGA T, Rat β-actin-R: GGA CAG TGA GGC CAG GAT AGA. The test was repeated three times for each sample.

### Statistical analysis

The data are expressed as the mean ± standard deviation. The normality test was performed using Shapiro Wilk (SPSS 22.0 software, Inc., Chicago, IL, USA), and p > 0.05 was considered to indicate a normal distribution. Differences between groups were measured by one-way analysis of variance. P < 0.05 was considered statistically significant.

## Results

### Curcumin reduced the deposition of kidney stones

**Figure 1:**
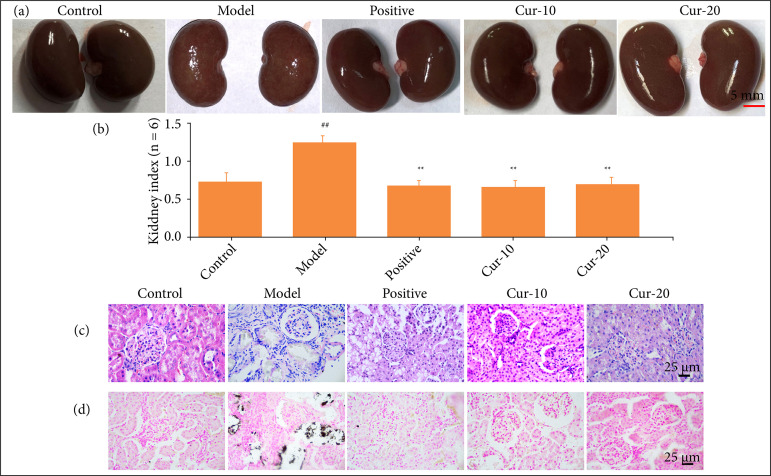
Curcumin reduces EG-induced calcium oxalate deposition. **(a)** Photographies of rat kidneys in each group. The kidney surface of the control was smooth without particle sensation, the kidney surface of the model was swollen, accompanied by stone like particles; **(b)** Kidney index (n = 6), ^**^P < 0.01 means comparison with model group, ^##^P < 0.01 means comparison with the control group. **(c)** HE staining results of kidney tissue, the black part indicates kidney stone; **(d)** von Kossa staining result of kidney tissue, the black part indicates calcium deposition. Their magnification is 400 ×.

In the rat kidney stone model, it was found that the kidney was swollen, and the surface was uneven, accompanied by stone-like particles (Fig. 1a). In the positive, control, Cur-10, and Cur-20 groups, the surfaces of kidneys were smooth with no significant difference compared with the control group. The kidney index showed that after the administration of the stone attractant, the kidney index in the model group was significantly higher than the other groups (Fig. 1b, P < 0.01). However, the renal index of rats in the curcumin treatment group was significantly decreased compared with the model group, and was not significantly different from that of the positive group (P > 0.05). In addition, the results of HE staining confirmed that EG caused kidney stones in rats, as indicated by the black part (Fig. 1 c,d). After curcumin treatment, there was no black deposition in the pathological stions of rat kidneys. The above data showed that curcumin could maintain the normal appearance of rat kidneys, and inhibit the formation of renal stones during the process of EG-induced kidney stones, which was equivalent to the efficacy of the positive drug potassium citrate in efficacy.

### Curcumin reduces renal calculi to the kidney

**Figure 2:**
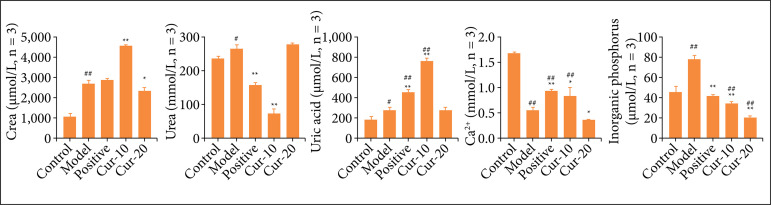
Urine biochemical test results. Changes of Cr, Ur, UA, Ca^2+^, and IP in urine (n = 3). The differences between two groups were measured by Student’s t-test. ^*^P < 0.05, ^**^P < 0.01 means comparison with model group, ^#^P < 0.05, ^##^P < 0.01 means comparison with the control group.

After EG treatment, the urinalysis results of rats in each group within 12 h showed that the Cr content in urine increased significantly (Fig. 2, P < 0.01). However, the positive control group and both Cur-10 and Cur-20 had no significant effect on the Cr content. Compared with the control group, the Ur level in model increased significantly after EG induction (P < 0.01), compared with model group, positive group and Cur treatment group could reduce Ur content (P < 0.05). The content of UA in model also increased significantly after EG induction, too (P < 0.01). Compared with the model group, Cur treatment was able to significantly reduce UA values (P < 0.05), although there were also some differences from the control group. In comparison, the content of Ca^2+^ was not significantly different among the groups (P > 0.05). Compared with the model group, curcumin treatment was able to significantly reduce IP values (P < 0.05), this is better than positive.

**Figure 3:**
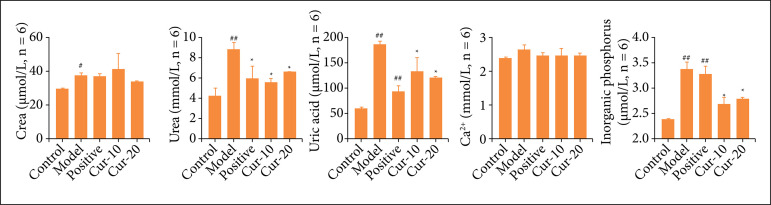
Serum biochemical test results. Changes of Cr, Ur, UA, Ca^2+^, and IP in serum (n = 6). The normality test was performed using Shapiro–Wilk, differences between two groups were measured by Student’s t-test. ^*^P < 0.05, ^**^P < 0.01 means comparison with model group, ^##^P < 0.01 means comparison with the control group.

The biochemical test of rat serum showed that compared with the control, the UA content in the other groups’ serum increased after the induction of the stone attractant (Fig. 3, P < 0.01). However, neither curcumin nor positive drug treatment reduced the content of UA. This result suggested that curcumin could not reverse EG-induced upregulation of serum UA levels in the serum. In the model group, the Ca^2+^ level was significantly higher than the Ca^2+^ level of the control. In contrast, the increase in Ca^2+^ was significantly alleviated after the administration of curcumin and the positive control drug, which was significantly different from the model group (P < 0.01). The IP content in serum was detected. Compared with the control, the serum IP content increased significantly in the positive, Cur-10, and Cur-20 groups (P < 0.01). In the model group, the IP content was significantly reduced (P < 0.01). Compared with that in the control group, the SOD content in serum was significantly decreased after the treatment with the EG (P < 0.01). Compared with the model, the SOD content in the positive, Cur-10, and Cur-20 groups was significantly increased (P < 0.05). Moreover, curcumin reduced the concentration of MDA in the serum. In this regard, curcumin has shown more advantages in reducing MDA than the positive control (potassium citrate).

### Curcumin reduced the damage of oxidative stress in rat kidneys

**Figure 4:**
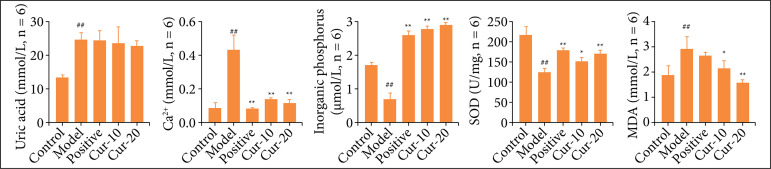
Kidney tissue biochemical test results. Changes of UA, Ca^2+^, IP, SOD and MDA in kidney tissue (n = 6). The normality test was performed using Shapiro–Wilk, differences between two groups were measured by Student’s t-test. ^*^P < 0.05, ^**^P < 0.01 means comparison with model group, ^#^P < 0.05, ^##^P < 0.01 means comparison with the control group.

The results of the renal biochemical test showed that compared with control, the Cr, Ur, and UA contents in the model increased significantly (Fig. 4, P < 0.01). Compared with the control, the Ca^2+^ in the model group was significantly reduced (P < 0.01). However, Cr did not change significantly in the positive and Cur groups. In addition, the UA content in renal tissue was instead increased in the positive and Cur groups. Positive drug and curcumin could significantly reduce the content of Ca^2+^ and IP (P < 0.01), and the effect of high concentration of curcumin was better than that of the positive control drug.

### Curcumin decreased the expression of OPN in rat kidneys

**Figure 5:**
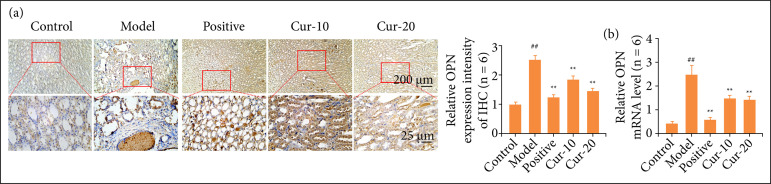
Detection of the expression of OPN in kidney tissue. **(a)** OPN immunohistochemical staining results (n = 6). Compared with the control group, the expression of OPN in the model group was obvious. The positive expression of OPN was in a small aggregate state; **(b)** Relative gene expression of OPN (n = 6). The normality test was performed using Shapiro–Wilk, differences between two groups were measured by Student’s t-test. ^*^P < 0.05, ^**^P < 0.01 means comparison with model group, ^##^P < 0.01 means comparison with the control group.

OPN is distributed in a variety of tissues and cells. It is the main protein in stone formation and plays an essential role in forming crystals^22^^,^ 23. This study found that the protein expression of OPN was mainly distributed in renal tubules and blood vessels, and the expression in the glomeruli was very shallow (Fig. 5a). Compared with the control group, the expression of OPN in the model group was obvious. It was particularly prominent in the calcium salt deposits, while the positive expression of OPN was in a small aggregate state. With different concentrations of curcumin treatments, it was found that the expression of OPN in the Cur-10 was still at a high level, and the positive reaction in the Cur-20 was significantly weakened. However, in the positive, Cur-10, and Cur-20 groups, none showed the large-scale concentrated expression expressions in the calcium deposits as model groups. Figure 5b shows the result of the OPN expression in kidney tissue. Compared with the control, the relative expression of the OPN gene in the model’s kidney was significantly increased (P < 0.01). OPN expression in the curcumin treatment groups was significantly reduced compared with that in the model after different doses of curcumin treatment (P < 0.01). However, there was no difference between the curcumin treatments (P > 0.05). The results showed that a low dose of curcumin could also efficiently inhibit OPN.

## Discussion

In this study, a rat model of kidney stones was successfully established through EG induction. Furthermore, considering that the absorption efficiency of curcumin in the human gastrointestinal tract was very low, most of it would be excreted through the intestine^24^. Therefore, we believe that the method of absorption through the intestinal tract has yet to be discussed, so this experiment used the method of subcutaneous injection at the back of the rat’s neck to administer the drug, and the method was innovative.

The HE staining results of kidney tissue sections showed that the model’s kidney tissue was severely expanded, tubules were destroyed, and epithelial cells had fallen off. Glomerular atrophy, necrosis, and sclerosis were visible, and there was a large infiltration of inflammatory cells. The renal tubules were dilated, with mesenchymal fibrosis, and large areas of kidney stone crystals. This result indicated that calcium oxalate kidney stones induced by EG could affect kidney function. We did not see obvious stones in the positive group. Cur-20 group tissue did not produce obvious lesions or stones. In Cur-10 tissue, the glomeruli had a small degree of atrophy but no expansion or disease. The results of von Kossa staining showed that the model had a large amount of calcium ion stone deposits at the skin and pulp junction. In contrast, no obvious calcium ion deposits were seen in the other groups. The black part in the picture was the explanation part. The renal function of rats with calcium oxalate stones was significantly impaired, and these results were consistent with the results of Li et al.^11^.

Biochemical analysis results showed that both potassium citrate gavage and different doses of curcumin injection could effectively reduce the IP in the urine. However, it was worth noting that the Ur concentration in the Cur-10 group was significantly lower than the Ur in Cur-20 group. Analysis of the calcium ion content in urine showed that the calcium ion content in the urine of rats after EG induction was significantly reduced, and the calcium ion content was inversely proportional to the curcumin dose. Potassium citrate is an energetic calcium ion chelating agent that can reduce calcium ion activity, concentration, and saturation, thereby reducing the formation of calcium salt crystals and achieving the effect of stone removal^25^^,^ 26. Combining the structural formula of curcumin and the experimental results, we speculated that the hydroxyl group in the curcumin diphenol structure could form a hydrogen bond with the carboxyl group of oxalate, reducing the concentration of free oxalate ions, reducing the deposition of calcium ions, and inhibiting the growth of calcium oxalate crystals.

An abnormal increase in IP content in serum is an essential indicator for detecting chronic kidney disease^27^^,^ 28. This study found that after injection of different doses of curcumin, the content of Ur, UA, and IP in the serum of the curcumin treatments decreased significantly, indicating that the curcumin drug had played a role in protecting renal function. Studies have pointed out that curcumin can promote the expression of SOD, the main active product downstream of the Nrf2 signaling pathway, by promoting the nuclear accumulation of Nrf2^29^^-^ 31, reducing the oxidative damage level and oxidative stress in the kidney, inhibiting the development of kidney stones, and helping to restore kidney function. In past reports, there have been studies on biochemical analysis. Here, we analyzed various indicators in serum, urine, and kidney to more intuitively reveal the specific effects of curcumin treatment to better reflect the efficacy of the drug.

The ROS generated by oxidative stress can stimulate cell apoptosis and autophagy, further aggravating cell damage, and SOD can alleviate tissue damage caused by ROS. Compared with the model, after different doses of curcumin and potassium citrate treatment, SOD activity was significantly increased. Furthermore, the MDA content of the model group decreased, and the MDA content of the Cur-10 and positive groups was significantly lower than that of the model.Cur-20 group had the lowest MDA content, which was extremely different from the model. The results showed that curcumin could inhibit the development of kidney stones by reducing the level of oxidative damage and oxidative stress in the kidney tissue, and protect kidney cells in a dose-dependent manner.

OPN plays a critical role in the formation of stones and is the leading organic component of urinary calcium kidney stones^23^^,^ 32. Moreover, after binding to the receptor CD44, it could promote calcium oxalate crystals in epithelial cells, which had been confirmed to be overexpressed in animal models of kidney stones induced by hyperoxaluria^33^. The results of immunohistochemical showed that OPN was concentrated in renal tubules and blood vessels, and the expression in glomeruli was very low. The positive expression of the OPN in the model group was stronger. There was a particularly strong positive expression in the calcium salt deposition site. In contrast, the expression of OPN in the curcumin treatments was significantly lower than that of model, indicating that curcumin could inhibit the gene expression of OPN and even inhibit the formation of kidney crystals.

## Conclusion

The subcutaneous injection of curcumin could alleviate the oxidative stress damage of calcium oxalate kidney stones induced by EG within a specific dose range. Curcumin may dissolve calcium oxalate stones in the renal tubules of rats and reduce the oxidative damage of calcium oxalate stones to the kidneys. Therefore, curcumin could be used as a new drug candidate to treat kidney stones.

## Data Availability

Data will be available upon request.
